# Remodelling of the fibre-aggregate structure of collagen gels by cancer-associated fibroblasts: A time-resolved grey-tone image analysis based on stochastic modelling

**DOI:** 10.3389/fimmu.2022.988502

**Published:** 2023-02-03

**Authors:** Cedric J. Gommes, Thomas Louis, Isabelle Bourgot, Agnès Noël, Silvia Blacher, Erik Maquoi

**Affiliations:** ^1^ Department of Chemical Engineering, School of Engineering, University of Liège, Liège, Belgium; ^2^ Laboratory of Tumor and Development Biology, GIGA-Cancer, University of Liège, Liège, Belgium

**Keywords:** fibrillar collagen, cancer, cancer-associated-fibroblasts (CAFs), confocal microscopy, image analysis, stochastic modelling, correlation functions, spheroid

## Abstract

**Introduction:**

Solid tumors consist of tumor cells associated with stromal and immune cells, secreted factors and extracellular matrix (ECM), which together constitute the tumor microenvironment. Among stromal cells, activated fibroblasts, known as cancer-associated fibroblasts (CAFs) are of particular interest. CAFs secrete a plethora of ECM components including collagen and modulate the architecture of the ECM, thereby influencing cancer cell migration. The characterization of the collagen fibre network and its space and time-dependent microstructural modifications is key to investigating the interactions between cells and the ECM. Developing image analysis tools for that purpose is still a challenge because the structural complexity of the collagen network calls for specific statistical descriptors. Moreover, the low signal-to-noise ratio of imaging techniques available for time-resolved studies rules out standard methods based on image segmentation.

**Methods:**

In this work, we develop a novel approach based on the stochastic modelling of the gel structure and on grey-tone image analysis. The method is then used to study the remodelling of a collagen matrix by migrating breast cancer-derived CAFs in a three-dimensional spheroid model of cellular invasion imaged by time-lapse confocal microscopy.

**Results:**

The structure of the collagen at the scale of a few microns consists in regions with high fibre density separated by depleted regions, which can be thought of as aggregates and pores. The approach developped captures this two-scale structure with a clipped Gaussian field model to describe the aggregates-and-pores large-scale structure, and a homogeneous Boolean model to describe the small-scale fibre network within the aggregates. The model parameters are identified by fitting the grey-tone histograms and correlation functions of the images. The method applies to unprocessed grey-tone images, and it can therefore be used with low magnification, noisy time-lapse reflectance images. When applied to the CAF spheroid time-resolved images, the method reveals different matrix densification mechanisms for the matrix in direct contact or far from the cells.

**Conclusion:**

We developed a novel and multidisciplinary image analysis approach to investigate the remodelling of fibrillar collagen in a 3D spheroid model of cellular invasion. The specificity of the method is that it applies to the unprocessed grey-tone images, and it can therefore be used with noisy time-lapse reflectance images of non-fluorescent collagen. When applied to the CAF spheroid time-resolved images, the method reveals different matrix densification mechanisms for the matrix in direct contact or far from the cells.

## 1 Introduction

In living tissues, cells are commonly embedded within complex networks of extracellular matrix (ECM) constituted of diverse highly cross-linked components, including fibrous proteins, proteoglycans, glycoproteins and polysaccharides (1–3). There are three classes of fibrous ECM proteins: collagen, elastin and fibronectin. Each of the individual ECM components exhibits specific biomechanical and biochemical properties that are related to its polymer structure, size and binding affinities to signaling molecules (4–7). Recent data have highlighted the importance of ECM topology (organization of fibres, matrix porosity and density), which influences the mechanical properties of the matrix (8, 9). For instance, aligned bundles of collagen increase matrix stiffness (4). Along with fibre organization, the degree of porosity of the ECM influences its rheological properties, which in turn modulates the cell behavior (proliferation, differentiation, survival and motility) (10). The ECM fibers are constantly remodeled by cells through synthesis, re-orientation, deformation and degradation (11).

Collagens containing 28 different types are the most abundant components of the ECM and their structure and composition differ across various tissue types (12–15). Type I collagen represents the most common fibrillar collagen in vertebrates with more than 90% of collagen molecules in the human body. Its synthesis, stiffening and remodelling are involved in physiological (wound healing, inflammation, tissue repair) and pathological (inflammation, fibrosis, cancer) processes (14, 16–18). Type I collagen gels have been used extensively as experimental models for ECM. They feature hierarchical structures that are self-assembled through multiple steps. At the molecular level, type I collagen first forms polypeptides. Three polypeptide chains wrap together into a triple helix, the tropocollagen, with an approximate diameter of 1.5 nm, and a length of 300 nm. Multiple tropocollagens bundle together into microfibrils and subfibrils, whose diameters are about 6 and 25 nm, respectively. The microfibrils and subfibrils further bundle into a collagen fibril, which can reach a length of up to 1 mm and hundreds of nanometers in diameter. The length and diameter of the generated fibers, and subsequently their mechanical property depend on several factors such as protein concentration, temperature or pH value. For example, Doyle et al. (19) found that gels formed at 37°C contained single fibrils of 310 nm in diameter, whereas gels formed at 21°C have fibers bundled by 2 to 12 fibrils. The resulting collagen matrix is a porous, disordered assembly of collagen fibers (20).

In normal tissues, the structure and mechanics of the ECM are tightly regulated *via* homeostasis between the ECM and resident stromal cells (21, 22). During tumorigenesis, deregulated biological processes impair this homeostasis, triggering aberrant ECM composition, architecture, and functions. The ECM abnormality reciprocally fuels the growth and metastasis of cancers, thereby causing a deadly positive feedback cycle (20). Many solid tumors such as those of the breast, lung and pancreas are characterized by the presence of a desmoplastic stroma typified by an accumulation of fibrillar collagen. This collagen accretion increases the stiffness and creates discrete structural patterns called tumor-associated collagen signatures (TACS). TACS classifies three different collagen shells of the invasive breast tumor, with TACS3, the outermost layer defined by linearized and bundled collagen fibers oriented perpendicularly to the tumor surface (23). Clinically, there is a well-established link between the collagen architecture of the primary tumor and prognosis. TACS3-positive patients had lower disease-free survival (24, 25). These anisotropic patterns modulate the migration of tumor cells but also fibroblasts, immune and endothelial cells in the tumor microenvironment (23, 26–29). It is noteworthy that aligned ECM fibers also lead to anisotropic diffusivity (30, 31). Hence, the transport of secreted factors and exosomes along the direction of ECM alignment is significantly enhanced, which modulate the cross-talk between different cell types (31).

Cancer-associated fibroblasts (CAF) are activated fibroblasts that make up the majority of the non-cancerous cells of the desmoplastic stroma (32, 33). They can promote primary tumor growth and metastatic dissemination through different mechanisms (34–36) including the release of cytokines, chemokines, growth and angiogenic factors [for recent review articles on the contributions of CAFs during cancer progression, see (37–39)]. CAFs synthesize, secrete, and assemble large amounts of type I, III, IV, V, and XII collagen (40, 41), hyaluronic acid (42), laminin (43), and fibronectin (44). At the same time, they degrade the nearby ECM by secreting proteases, including matrix metalloproteinases (45, 46), thus remodeling the local tumor microenvironment (TME) by promoting tissue hardening and stromal cell fibrosis (47). The formed scaffold structure provides a permissive environment for the interaction between cells and cytokines, increasing the invasion of cancer cells (48). It also forms a physical barrier between cancer cells and immune cells as well as therapeutic drugs, thereby leading to tumor immune evasion and drug resistance (49). For example, the rigid ECM surrounding solid tumors prevents the cytotoxic CD8+ T cells from accessing the cancer cells (50). In a similar way, aligned fibronectin and collagen fibers located around lung tumor epithelial cell regions dictate the migratory trajectory of T cells and restrict them from entering tumor islets (51). Recently, two sub-populations of CAFs have been shown to orchestrate a particular structural tissue organization through dense and aligned fiber deposition, promoting T cell exclusion in human lung tumors (52). As a result, tumor cells can evade the immune defense even when these T cells have been activated. Collagens present in the TME also affect the function and phenotype of various types of tumor-infiltrating immune cells such as tumor-associated macrophages and T cells, suggesting that tumor-associated collagen could have important immune modulatory functions within the TME, affecting cancer progression as well as the efficacy of cancer immunotherapy (53). Consequently, CAFs and the associated ECM are intimately associated with the progression of cancers, the immune response and the prognosis of patients (54–56).

Understanding how CAFs influence the architecture of collagen is key to improving our understanding of cancer cell invasion through a dense collagenous stroma as observed in breast, lung and pancreatic cancers. At macroscopic scales, this can be studied with gel-contraction assays (57–59) and bead tracking (60–63), whereby the mechanical deformation of the collagen matrix is monitored without having to explicitly consider the underlying remodelling of the collagen network. More detailed understanding of the process, based on local influence of individual cells on the matrix and the biochemical interactions involved can be obtained using atomic-force and confocal microscopy at the scale of the cells (64). However, few studies have attempted to reconcile the macroscopic and cellular approaches to characterize the influence of cells on collagen architecture.

Different non-disruptive techniques based on light microscopy are available to dynamically image 3D cell-populated collagen gels (14). Among these techniques, second harmonic generation (SHG) microscopy represents one of the most widely used technique for imaging fibrillar collagens. Besides SHG, confocal reflection microscopy (CRM) is a high-resolution technique for imaging specimens that differ in refractive index from their surroundings or which possess a high reflectance (65). It is widely used to visualize polymers and biomaterials. A laser-scanning confocal microscope in reflection mode is able to detect variations of backscattered light intensity at the collagen-to-media interface for each sequential focal plane, resulting in the reconstruction of a three-dimensional image of the sample (66). CRM is readily applicable to observing gels in which simultaneous, multimodal imaging of collagen (with CRM) and cells (with confocal fluorescence microscopy, CFM) is carried out to investigate cell-environment interactions (66–68). This methodology offers a fundamental advantage over collagen imaging with CFM in that the collagen network need not be labeled with fluorophores, which potentially interfere with the polymerization process (69). In contrast with CFM, CRM can only detect fibers that are oriented roughly horizontally to the imaging plane. This creates a “blind spot” as CRM misses the vertical fibers. Therefore, collagen networks imaged with CRM appear anisotropic, and fewer fibers are visible when compared with CFM. Such blind-spot effects are also observed with SHG imaging (70).

Developing image-analysis tools to dynamically characterize the collagen-fibre network and its space dependent microstructural modifications in the context of cellular migration is challenging for a variety of reasons. In particular, the low signal-to-noise ratio of imaging techniques suitable for time-resolved studies at the required resolution precludes standard image analysis methods based on the segmentation of the fibrillar structures (71–73). Grey-tone image analysis methods have been developed in this context. Many of these studies focus on the analysis of fibre orientation using a variety of methods such as pixel-wise gradient estimations (74), Fourier transforms (75), mathematical morphology (76), or orientation-index analysis (77). In addition to fibre orientation, another important aspect of ECM remodelling concerns the spatial distribution of the fibres. The spatial heterogeneity the collagen network is known to be a key determinant of the collagen mechanical properties (20, 70, 78). It has also been reported using grey-tone co-occurrence matrix analysis and correlation functions that the collagen becomes less-homogeneous in the course of melanoma development (77), as well as epithelial (79, 80) tumor progression. In these studies, however, correlation functions are used as general tools for textural characterization, without any microstructural interpretation in terms of collagen fibres. General image analysis methods that can be used to quantitatively characterize the ECM heterogeneity and statistically capture geometrically complex fibre densification patterns, are yet to be developed.

The goal of the paper is to address this issue and develop such a method. The multidisciplinary approach we propose here is based on the stochastic modelling of grey-tone fibre structures in CRM images, and on the identification of the model parameters from their correlation functions and grey-tone histograms. In order to develop and validate the methodology, acellular collagen gels of different concentrations are considered first, and imaged by both CFM and CRM. The potential of the method is illustrated afterwards by analysing the ECM remodelling in the context of a collagen-embedded CAF spheroid model, and dynamically analyzing the space-dependent evolution of the fibre network that accompanies cellular migration.

## 2 Materials and methods

### 2.1 Materials

Dulbecco’s modified Eagle medium (DMEM), L-glutamine, sodium pyruvate, penicillin, streptomycin and 0.25% trypsin/EDTA solution were purchased from ThermoFisher Scientific. Recombinant platelet-derived growth factor BB isotype (PDGF-BB) was obtained from R&D Systems. Fetal bovine serum (FBS), sodium bicarbonate, and 10× concentrated DMEM, and high viscosity carboxymethylcellulose sodium salt were obtained from Sigma-Aldrich. High concentration acid soluble native non-pepsinized type I rat tail collagen was purchased from Corning and DQ-Collagen™type I from bovine skin fluorescein conjugate was obtained from ThermoFisher. SPY650-DNA, a non-toxic, cell permeable and highly specific live cell DNA probe was purchased from Spirochrome.

### 2.2 Preparation of collagen gels

Type I collagen gels were prepared for imaging of collagen architecture and spheroid invasion assays. Collagen gels of 2.0 mg/mL and 3.0 mg/mL were prepared by diluting the stock collagen solution (8-11 mg/mL) with 10× concentrated DMEM, NaHCO_3_, 1N NaOH, milliQ H_2_O and neutralized to pH 7.2. To visualize collagen fibers by CFM, fluorogenic DQ-collagen I was mixed with unlabeled diluted collagen as previously described (81), obtaining a final concentration of 20 µg/mL. Collagen dilutions were performed and maintained on ice until use. Droplets (25 µL) of diluted collagens were spotted in 35mm-glass bottom *μ*-dishes (Ibidi) and polymerized during 45 min at 19°C, followed by a 30 min incubation at 37°C. The polymerization temperature of 19°C was chosen to provide a matrix architecture comparable to *in vivo* with a network consisting of a mixture of a few thin bundles and many thick bundles (82). After completion of collagen polymerization, 1 mL of preheated DMEM supplemented with 5% FBS was added to the dishes. Image acquisition was performed after 24 h of incubation at 37°C with 5% CO_2_.

### 2.3 Cell culture

Mouse CAFs (CAFs) have been isolated from mammary gland tumors of mammary specific polyomavirus middle T antigen overexpression mouse model (FVB/N-Tg(MMTV-PyVT)634Mul/J) at 12 weeks as previously described (83) and immortalized with the pLenti HPV16 E6-E7 RFP, expressing a cytoplasmic red fluorescent protein (RFP).

Briefly, tumour samples were cut into small pieces and enzymatically digested with a collagenase solution for 45 min at 37°C. After filtration and centrifugation of cell suspension, the pellet was washed, resuspended, and cultured in high glucose DMEM supplemented with FBS (10%), L-glutamine (2 mM), penicillin (100 U/mL), and streptomycin (100 µg/mL). Cells were plated into plastic culture plates for 30 min to let CAFs adhere. The supernatant containing tumor cells was then removed (CAFs adhere more rapidly to tissue culture plates than epithelial cells). When the plates were nearly confluent, a differential trypsinization was performed to eliminate the contaminating epithelial cells (CAFs were efficiently detached from the bottom of the plate, whereas epithelial cells remained attached).

To rule out that these cells were derived from cancer cells undergoing an epithelial-to-mesenchymal transition, an RT-qPCR was performed to detect the mRNA coding for the polyoma middle T (PyMT) oncogene (specifically expressed by the epithelial cells of the MMTV-PyMT mice). While epithelial PyMT-derived cancer cells are expressing the PyMT oncogene mRNA, the CAF populations used in this study were completely negative (data not shown). The expression of different markers of CAFs including fibroblast activated protein *α* (FAP), *α* -smooth muscle actin (ACTA2), tenascin C (TNC), collagen type 1 (COL1A1), collagen type 3 (COL3), PDGFRB, and *α*11 integrin (ITGA11) was also quantified by RT-qPCR. Higher expression levels for these different mRNAs were measured in CAFs when compared with cancer cells (**Supplementary Materials, Figure S1**). Cultures were maintained at 37°C with 5% CO_2_ until their confluence reaches about 80%.

### 2.4 Plasmids, lentiviral vectors generation and cell transduction

Gene transfer lentiviral plasmids was purchased from Vector Builder Company (#VB151006-10035): pLenti HPV16 E6-E7 RFP (Puro). This plasmid allows HPV16 E6/E7 fusion protein (https://www.ebi.ac.uk/ena/browser/view/ACI43214), mRFP expression, and selection marker (puromycin) expression driven by EF1a or PGK promoter, respectively. Lentiviral vectors were generated by the GIGA Viral Vectors platform. Briefly Lenti-X 293T cells (Clontech™, 632180) were co-transfected with a pSPAX2 (Addgene™, Cambridge, MA, USA) and a VSV-G encoding vector (84). Viral supernatants were collected 48h, 72h, and 96h post-transfection, filtrated (0.2 µM), and concentrated 100× by ultracentrifugation. The lentiviral vectors were then titrated with qPCR Lentivirus Titration (Titer) Kit (ABM™, LV900, Richmond, BC, Canada). CAFs were transduced with lentiviral vectors (30 TU/cell). Transduced cells were selected with 10 µg/mL puromycine (InvivoGen). The absence of RCL and mycoplasma in cell supernatant was confirmed with qPCR Lentivirus Titration kit and MycoAlert™PLUS Mycoplasma Detection Kit (Lonza, LT07-710), respectively.

### 2.5 Spheroid invasion assay

Spheroids were prepared by seeding 1000 CAFs in 100 µL of spheroid formation medium composed of 0.22 µm-filtered DMEM medium supplemented with 10% FBS and 20% carboxymethylcellulose 4000 centipoise. Cells were seeded in round-bottom non-adherent 96-well plates (CELLSTAR, Greiner Bio-One) and centrifugated at 1000 rpm for 5 min. Plates were incubated at 37°C with 5% CO_2_ for 48 hours to promote spheroid formation. The content of each well was transferred in a petri dish with a 200 µL pipette (with cutted tip) and individual spheroids were collected under a binocular microscope with a 10 µL pipette. Each spheroid was resuspended in 23 µL of diluted collagen (2 mg/mL) and spotted as a 25 μL drop in a prechilled 8 well-glass bottom chamber slide (Ibidi). The slides were then transferred immediately to 19°C and flipped to maintain the spheroids in the middle of the collagen drop (preventing their sedimentation to the glass surface or to the collagen/air interface). The extent of collagen polymerization and spheroid positioning were carefully controlled by microscopic examination throughout the polymerization step. After 30 min at 19°C, the slides were transferred at 37°C to complete the polymerization. Preheated culture medium (300 µL/well) supplemented with 5% FBS, 10 ng/mL PDGF-BB and SPY650-DNA (1000-fold dilution) was added to the slides and time-lapse imaging was initiated within an hour after collagen polymerization.

### 2.6 Confocal microscopy imaging

Images of the three-dimensional (3D) collagen gels were acquired with an inverted confocal laser scanning microscope (LSM 880 Airyscan Elyra S1, Zeiss) with a Plan-Neofluar 10× /0.30 N.A. or a Plan-Neofluar 20× /0.50 N.A. objective (Zeiss). The DQ-collagen containing gels were excited with 488 nm laser. Non-fluorescent collagen gels were imaged by CRM in Airyscan high-resolution mode with a simultaneous excitation of the matrix by 488 nm and 633 nm lasers. To avoid edge effects, images were acquired at least 100 µm away from the gel border, avoiding regions close to the gel/glass and gel/medium interfaces. To visualize collagen fibres of acellular gels (gels containing no cells), samples were imaged both by CFM and CRM, using the 20× objective. The resulting images have dimensions of 1000 × 1000 × 90 voxels with anisotropic voxel size of 0.42 × 0.42 × 1.10 µm^3^, corresponding to a physical volume of approximately 500 × 500 × 100 µm^3^.

Time-lapse imaging of spheroid-containing collagen gels was performed using the 10× objective with the samples incubated at 5% CO_2_ and 37°C in the on-stage incubator (Okolab). The collagen matrix was imaged by CRM in Airyscan high resolution mode with a 1.4× digital zoom (scaling per pixel: 0.59 µm × 0.59 µm × 3.29 µm) and CAF were imaged by CFM in Fast Airyscan mode (scaling per pixel: 0.91 µm × 0.91 µm × 3.29 µm). CAF images were rescaled to fit the images of the collagen matrix. CAF-derived RFP was excited by a 561 nm laser and detected at 591 nm. The SPY650-DNA nuclear stain was excited by a 633 nm laser and the signal was captured at 654 nm. The imaging of the spheroids and of the matrix required a sequential imaging of the same tridimensional zone, leading to two 3D image files. Images were recorded every 30 min up to 16 h. 3D stacks were obtained at a step size of 2 µm intervals. Type I collagen fibrils have a diameter ranging from 20 nm to several hundred nm (22) while fibres are larger in diameter. Given that the size of each pixel is 0.42 and 0.59 µm (for 20× and 10× objectives, respectively), it is not possible to distinguish fibrils from fibres (85), therefore the term “fibres” was used to include both fibrils and fibres.

The raw images were converted using the Airyscan algorithm from the Zeiss Zen Black software. The images were then subjected to a histogram stretching and converted to tiff format through the Zeiss Zen Blue software. This operation turned the original 16-bit images into 8-bit images, which were corrected by histogram adjustment to maximize the conservation of valuable information.

## 3 Results

### 3.1 Covariance and grey-tone correlation function

We first analyzed the structure of acellular type I collagen gels of different concentrations. To allow a direct comparison of the images generated by CRM and CFM, native unlabelled collagen was mixed with fluorescently-labelled collagen and polymerization was performed at 19°C to provide a matrix architecture comparable to *in vivo*, with a network consisting of a mixture of a few thin bundles and many thick bundles (19, 69, 86, 87).

**Figure 1:**
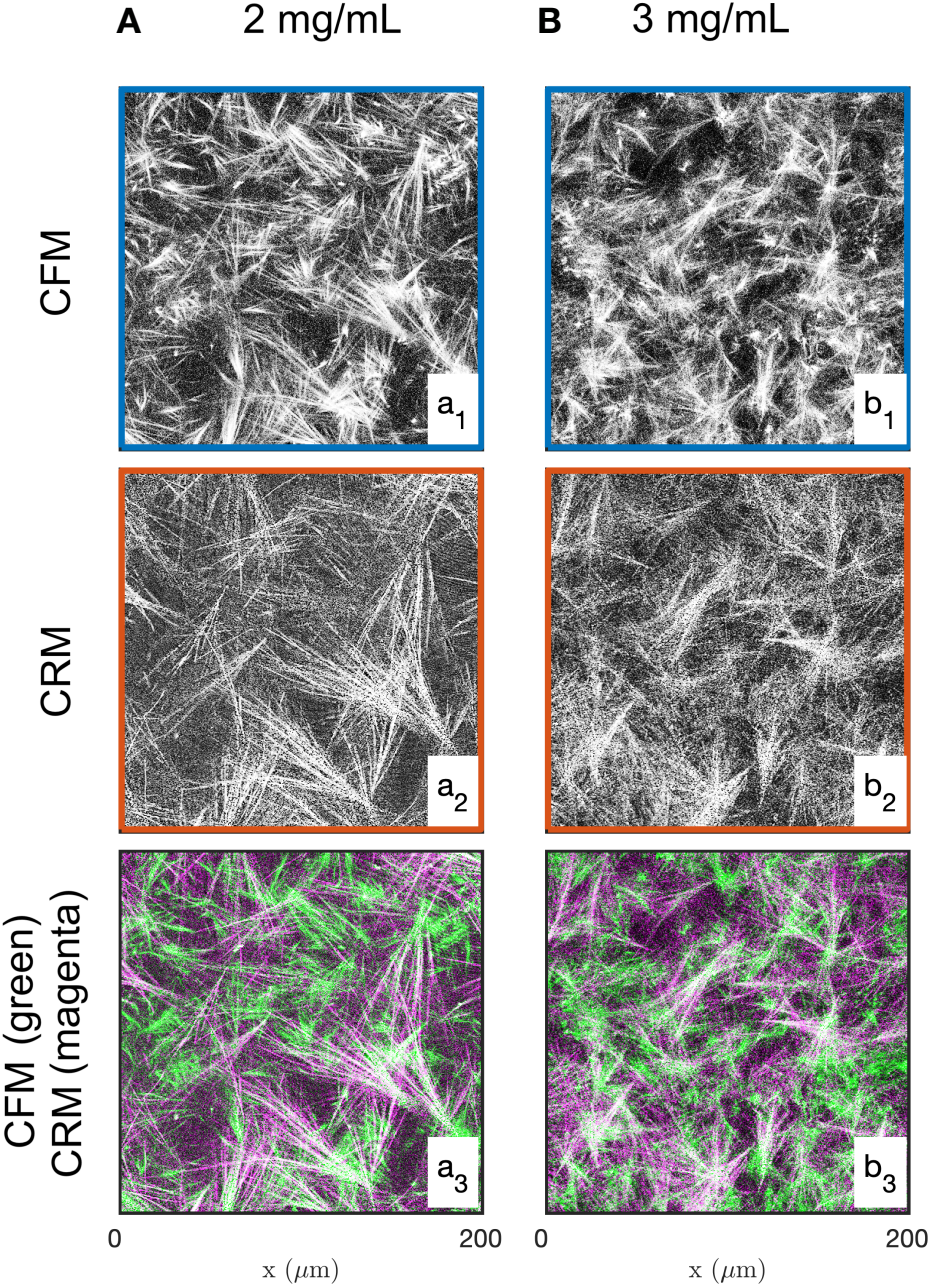
Images of the 2 mg/mL **(A)** and 3 mg/mL **(B)** acellular gels obtained by CFM **(a1, b1)** and CRM **(a2, b2)**. The bottom row **(a3, b3)** displays fused images with fluorescence and reflectance data in green and magenta, respectively.

Examples of images of the diluted (2 mg/mL) and concentrated (3 mg/mL) collagen gels obtained through CFM and CRM are given in **Figure 1**. These are 2D single-Z images taken out of the 3D images. The structural analysis is based on 15 images such as presented in the figure, for each gel and each imaging mode. Type I collagen fibrils have a diameter ranging from 20 nm to several hundred nm (88) while fibres are larger in diameter. Given that the size of each pixel is 0.42 and 0.59 µm (for 20x and 10x objectives, respectively), it is not possible to distinguish fibrils and fibres (85), therefore the term “fibres” was used to include both fibrils and fibres. The most salient structures are the fibre aggregates that are a few tens of micrometers across. The fused images in **Figures 1a3, b3** show that there is limited overlap between the CRM and CFM microscopy data: the fibres and aggregates are well captured in CFM but it is mostly the aggregates that are visible in the CRM data. Moreover, CRM is known to detect mostly fibres within the imaging plane and miss the vertical fibres (70).

**Figure 2:**
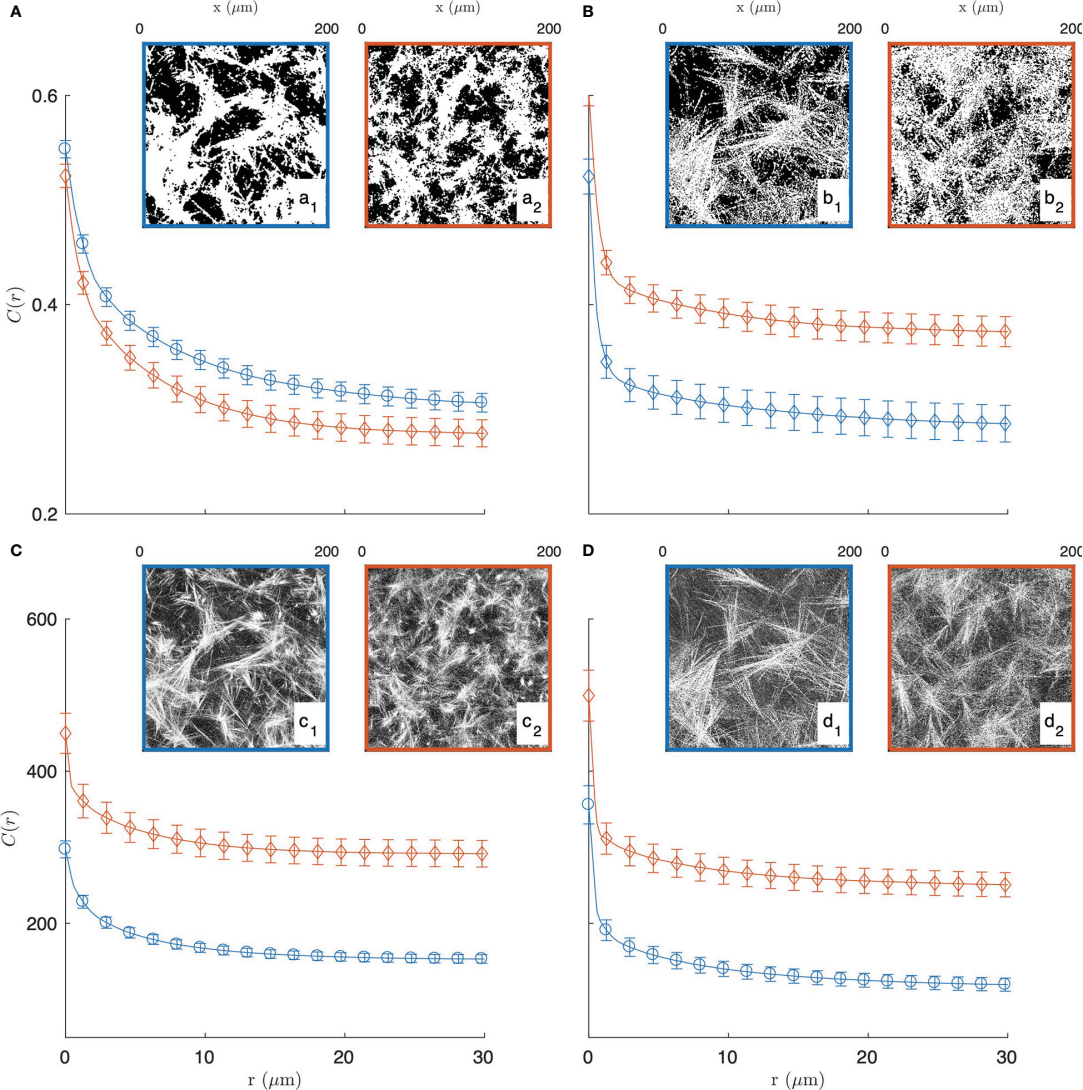
Experimental covariance (top) and grey-tone correlation function (bottom) of the 2 mg/mL (blue circle, **a1, b1, c1, d1**) and 3 mg/mL (red diamond, **a2, b2, c2, d2**) gels, imaged in fluorescence **(A, C)** and reflectance **(B, D)** modes. The insets display one of the fifteen images used for each condition, and the error bars are the standard errors of the mean.

In order to justify the need for the novel methods we propose in this paper, we first explored a standard image-analysis method based on image segmentation. In that spirit, the grey-tone images of the gels are converted to binary images following a method described in the **Supplementary Material** (see **Figure S2**). Examples of segmented images of the gels, with collagen in white and the rest in black, are provided in the insets of **Figures 2A, B**.

The segmented gel images display complex and disordered structures, which are also corrupted by noise in the case of the reflectance images (**Figures 2b1, 2b2**). In this context, the covariance - which describes the spatial correlation between all pixels intensities in the images (89–92)- was used to quantitatively analyze the gel structures. The covariances shown in **Figures 2A, B** were obtained from fifteen images taken in the same gel with dimension 200 × 200 µm^2^ as illustrated in the insets, *via* a Fourier-transform algorithm, and the error bars are the standard errors of the means.

The covariance *C*(*r*) of say, the white component of an image, has a clear geometrical interpretation as it is defined as the probability that any couple of points at distance *r* from one another both belong to that phase. For very small values of *r*, this definition coincides with the probability for a single point to belong to the white phase, which is numerically equal to its density *ϕ*_1_ normalized between 0 and 1. In the opposite limit, that is for very large distances *r* the covariance converges to the value $\phi_{1}^{2}$ , which corresponds to a horizontal asymptote in **Figure 2**. The shape of the covariance curve between those two limits characterizes the structures present in the images. In particular the progressive decrease of *C*(*r*) over distances of a few tens of microns testifies to the presence of structures with those dimensions, which we qualitatively referred to earlier as fibre aggregates.

The covariance of the fluorescence images of the gels (in **Figure 2A**) highlights at once the unsuitability of image segmentation in the present context. The covariance of the 2 mg/mL gel is found to be larger than that of the 3 mg/mL gel for any *r*, which means that densities of the segmented images contradict the actual collagen concentrations of the gels. This results from the fact that collagen-rich areas of the images display a variety of grey-tones related to the local fibre density (see **Figure S2B**), which information is lost during the all-or-nothing segmentation procedure. This general observation calls for grey-tone image analysis methods that preserve the structural information in the images. In that spirit, the grey-tone correlation functions *C*(*r*) of the gels are shown in **Figures 2C, D**. The latter are measured directly on the unprocessed images and they characterise the statistical correlation between grey-tones of all pixels that are at distance *r* from one another. In the case where the images contain only the values 0 and 1, this definition is mathematically equivalent to the covariance. We postpone to a later section the discussion of the structural significance of the grey-tone correlation function, but we already notice at this stage that the grey-tone data in **Figures 2C, D** scale with the actual collagen concentration of the 2 mg/mL and 3 mg/mL gels as they should.

### 3.2 Structural models

Covariance and grey-tone correlation functions convey indirect yet very rich structural information (93–95), which can notably be retrieved using structural models. We here present two models aimed at extracting structural information from the covariance data in **Figure 2**, which we generalize later to grey-tone correlation functions. In order to cope with the disordered structure of the gel, the models have to be stochastic (91, 92, 96, 97).

When using stochastic models, a structure is defined through probabilistic rules and this calls for specific concepts. In particular, it is convenient to introduce the indicator function of the structure *ℐ*(**x**), which takes the value 1 if the point **x** belongs to the structure and 0 otherwise (91). With such definition, the density of the model is calculated as


(1) $$\phi_{1}=\langle I\left(\text{x}\right)\rangle$$

where the brackets 〈〉 stand for the average value, calculated either over **x** or over all the possible realization of the model. Similarly, the covariance is calculated as the following two-point average


(2) $$C_{11}\left(r\right)=\langle I\left(\text{x}\right)I\left(\text{x}+\text{r}\right)\rangle$$

because the product ℐ(**x**)ℐ(**x**+**r**) is equal to one, only if the points **x** and **x**+**r** belong to the structure. In the latter equation, we have assumed statistical isotropy so that the dependence is only through the modulus *r* = |**r**|.

#### 3.2.1 Homogeneous fibre model

**Figure 3:**
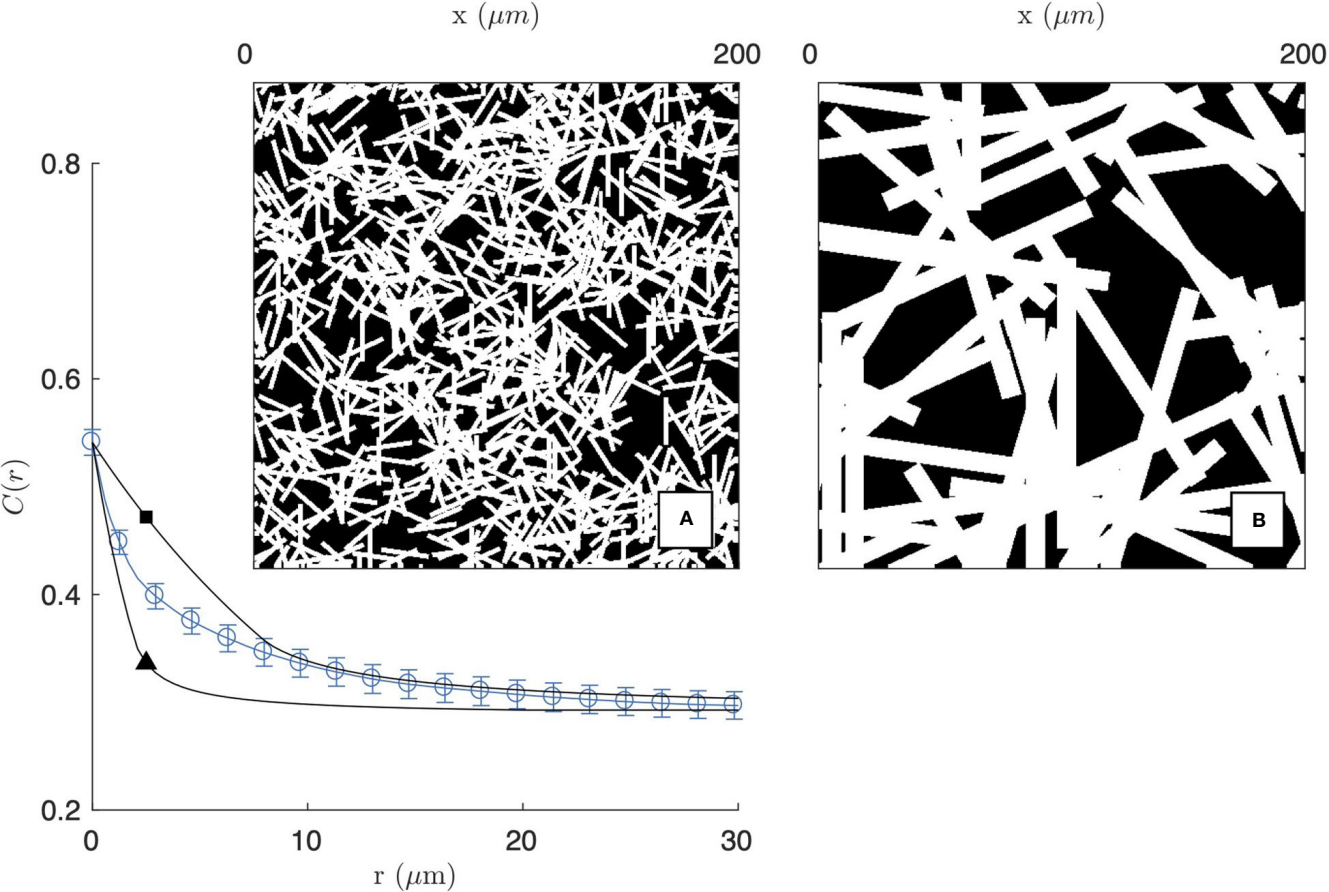
Homogeneous fibre model, obtained as a Boolean model of randomly oriented rectangles. The two realisations are obtained with length and diameter *L*_F_ = 20 µm, *D*_F_ = 1 µm (**A** and ▲) and *L*_F_ = 100 µm, *D*_F_ = 10 µm (**B** and □), and the calculated covariances are compared with that of the 2 mg/mL gel imaged by CFM (same as **Figure 2a1**).

The simplest model we consider to analyze the structures in **Figure 1** assumes that the gel matrix is statistically homogeneous. The model consists in tossing fibres (modelled as elongated rectangles) with random position and orientation, as sketched in **Figure 3**. Such model is described by three parameters, namely: the number density of fibres *θ* (unit µm^-2^), as well as their length *L*_*F*_ and diameter *D*_*F*_ (both in units of µm). Note that the spatial homogeneity of the probabilistic rules of the model does not preclude the existence of aggregates, which form when a large number of fibres coincidentally fall in the same region of space. The same holds for pores in the gel matrix.

The homogeneous fibre model is a particular case of a Boolean model (89, 92), for which the density and covariance are known analytically. In particular, the fibre density is


(3) $$\phi_{F}=1-\exp\;\left[-\eta \right]$$

where *η*=*θD*_*F*_ *L*_*F*_ is the density one would expect in absence of fibre overlap. The covariance is given by the following expression


(4) $$C_{FF}\left(r\right)=2\phi_{F}-1+{\left(1-\phi_{F}\right)}^{2}\exp\;\left[\eta K_{F}\left(r\right)\right]$$

where *K*_*F*_(*r*) is the geometrical covariogram of the randomly-oriented fibres, which is calculated as


(5) $$K_{F}\left(r\right)=\frac{2}{\pi }\int_{0}^{\pi /2}\left[1-\frac{r}{D_{F}}\sin\;(t)\right]\left[1-\frac{r}{L_{F}}\cos\;(t)\right]\text{d}t$$

for *r*<*D*_*F*_ and the upper integration bound is replaced by asin(*D*_*F*_/*r*) for *r*≥*D*_*F*_.

The covariance of the homogeneous fibre model is plotted in **Figure 3** for two fibres sizes. For the purpose of illustration, the model is compared with the experimental covariance of the segmented image of the 2 mg/mL gel (from CFM). In the figure, the number of fibres *θ* is chosen to achieve a density *ϕ*_*F*_≃0.54 comparable to the segmented image. As a consequence, the asymptotic values of *C*_*FF*_(*r*) are a close match to the gel covariance both for *r*=0 and for *r*→*∞*. The shape of the covariance at intermediate distances, however, cannot be captured by the homogeneous model. The model can account for either the small- or large-*r* data but not the two simultaneously. In the former case, the model captures the small-scale structure of the gel (**Figure 3A**) but it is unable to account for the aggregates, which are found to be more prevalent than what can be expected from randomness alone. In the latter case, the large-scale structure of the gel is reasonably reproduced, but this is done by replacing fibre aggregates by unrealistically large rectangles (**Figure 3B**).

#### 3.2.2 Fibre aggregates model

The inability of the homogeneous fibre model to reproduce the experimental covariance of gels proves that structures larger than individual fibres are more frequent than what can be accounted for by statistical fluctuations alone. The existence of such large pores and aggregates is notably apparent when comparing the realization of the homogeneous model in **Figure 3A** with the insets of **Figure 2**.

To address this issue, we introduce a second model that builds on two distinctly different structures. At the smallest scale the structure is assumed to be that of homogeneously-distributed fibres, identical to **Figure 3**, corresponding to indicator function ℐ_F_(**x**). The larger-scale structure, however, is accounted for by creating the indicator function of the entire structure ℐ(**x**) through the following multiplication


(6) $$I\left(\text{x}\right)={ℐ}_{F}\left(\text{x}\right)\times {ℐ}_{A}\left(\text{x}\right)$$

where ℐ_A_(**x**) is the indicator function of the aggregates, equal to one if **x** is inside an aggregate. Mathematically, Eq. (6) is equivalent to starting with a homogeneous fibre structure and subsequently carving pores out of it, using ℐ_A_(**x**) as a mathematical cookie-cutter.

Independently of the specific models chosen for ℐ_F_(**x**) and *ℐ*_*A*_(**x**), evaluating the average value of Eq. (6) yields the following density for the two-scale structure


(7) $$\phi_{1}=\phi_{F}\phi_{A}$$

where *ϕ*_*A*_ is the density of the aggregates, and *ϕ*_*F*_ is the density of the fibres within the aggregates. Equation (7) results from the general definition of the density in Eq. (1), with the assumption that the fibre and aggregate models are statistically independent from one another. The same assumption provides the following relation for the covariance of the solid phase


(8) $$C_{11}\left(r\right)=C_{FF}\left(r\right)C_{AA}\left(r\right)$$

as a consequence of Eq. (2).

For the small-scale structure, we assume the same fibre model as considered earlier, with density *ϕ*_*F*_ and covariance *C*_*FF*_(*r*) given in Eqs. (3) and (4). As the aggregates are very disordered with no well-defined shape, we model them with a clipped Gaussian-field approach approach (97–99) as described in the **Supplementary Material**. The two parameters of the large-scale model are the density of the aggregates *ϕ*_*A*_ and a single characteristic length *L*_*A*_ that controls their size.

**Figure 4:**
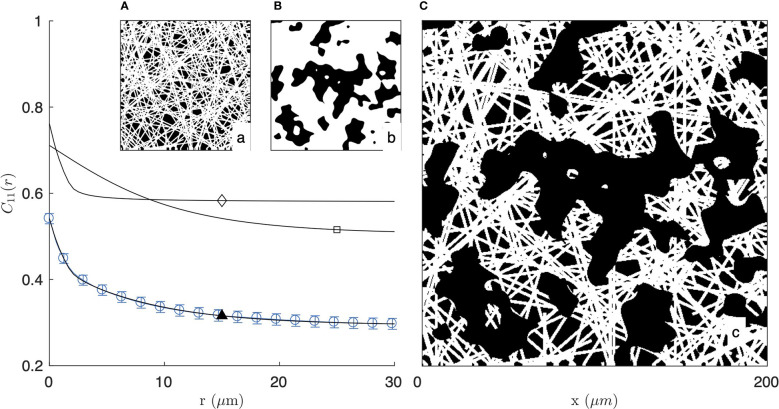
Covariance of the fibre-aggregate model, with homogeneous fibre model at small scale (**A** and ⋄) and clipped Gaussian-field model for the large-scale aggregates (**B** and □), combined to yield a two-scale structure (**C** and ▲). The calculated covariance is compared with that of the 2 mg/mL gel imaged by fluorescence microscopy (same as **Figure 2a1**).

As illustrated in **Figure 4** for the segmented image of the 2 mg/mL gel in fluorescence mode, the fibre-aggregate model captures well the experimental covariance of the gels. For the fitting of the data, the parameter *L*_*F*_ in the fibre model is irrelevant as the actual length of the fibres is controlled by the size of the aggregates. Equation (5) was therefore simplified to its long-fibre limit, namely *L*_*F*_→*∞*. The values of the remaining parameters are gathered in **Table 1** for the two gels and the two imaging modes. From the fitted parameters *ϕ*_*F*_ and *ϕ*_*A*_ of the fibre-aggregate model, the total density of the fibres *ϕ*_1_ was calculated through Eq. (7) and is also reported in **Table 1**.

**Table 1:** Structural parameters of the gels, obtained from fitting the covariance (binary images) or the correlation function and histogram (grey-tone images) with the fibre-aggregate model.

Imaging	Processing	Gel	*ϕ*_*F*_ (-)	*ϕ*_*A*_ (-)	*D*_*F*_ (µm)	*L*_*A*_ (µm)	*ϕ*_1_ (-)
CFM	Binary	2 mg/mL	0.76 ± 0.03	0.72 ± 0.01	1.9 ± 0.2	8.2 ± 1	0.55 ± 0.01
		3 mg/mL	0.76 ± 0.01	0.69 ± 0.01	1.5 ± 0.1	6.3 ± 0.4	0.52 ± 0.01
	Grey-tone	2 mg/mL	0.73 ± 0.04	0.32 ± 0.05	1.9 ± 0.1	6.6 ± 1	0.23 ± 0.03
		3 mg/mL	0.78 ± 0.06	0.49 ± 0.04	2.3 ± 0.1	7.7 ± 1	0.38 ± 0.06
CRM	Binary	2 mg/mL	0.60 ± 0.02	0.88 ± 0.04	0.7 ± 0.09	15 ± 1	0.53 ± 0.04
		3 mg/mL	0.67 ± 0.01	0.90 ± 0.02	0.90 ± 0.03	13 ± 1	0.61 ± 0.01
	Grey-tone	2 mg/mL	0.78 ± 0.05	0.41 ± 0.03	0.98 ± 0.2	7.6 ± 0.5	0.32 ± 0.01
		3 mg/mL	0.61 ± 0.06	0.56 ± 0.01	0.62 ± 0.1	14 ± 7	0.34 ± 0.02

*ϕ*_F_ , D_F_ : density and diameter of the fibres; *ϕ*_A_ , L_A_ : density and size of the aggregates; *ϕ*_1_ : total fibre density. The error bars are the standard deviations observed from the fits of the fifteen images in each condition.

**Figure 5:**
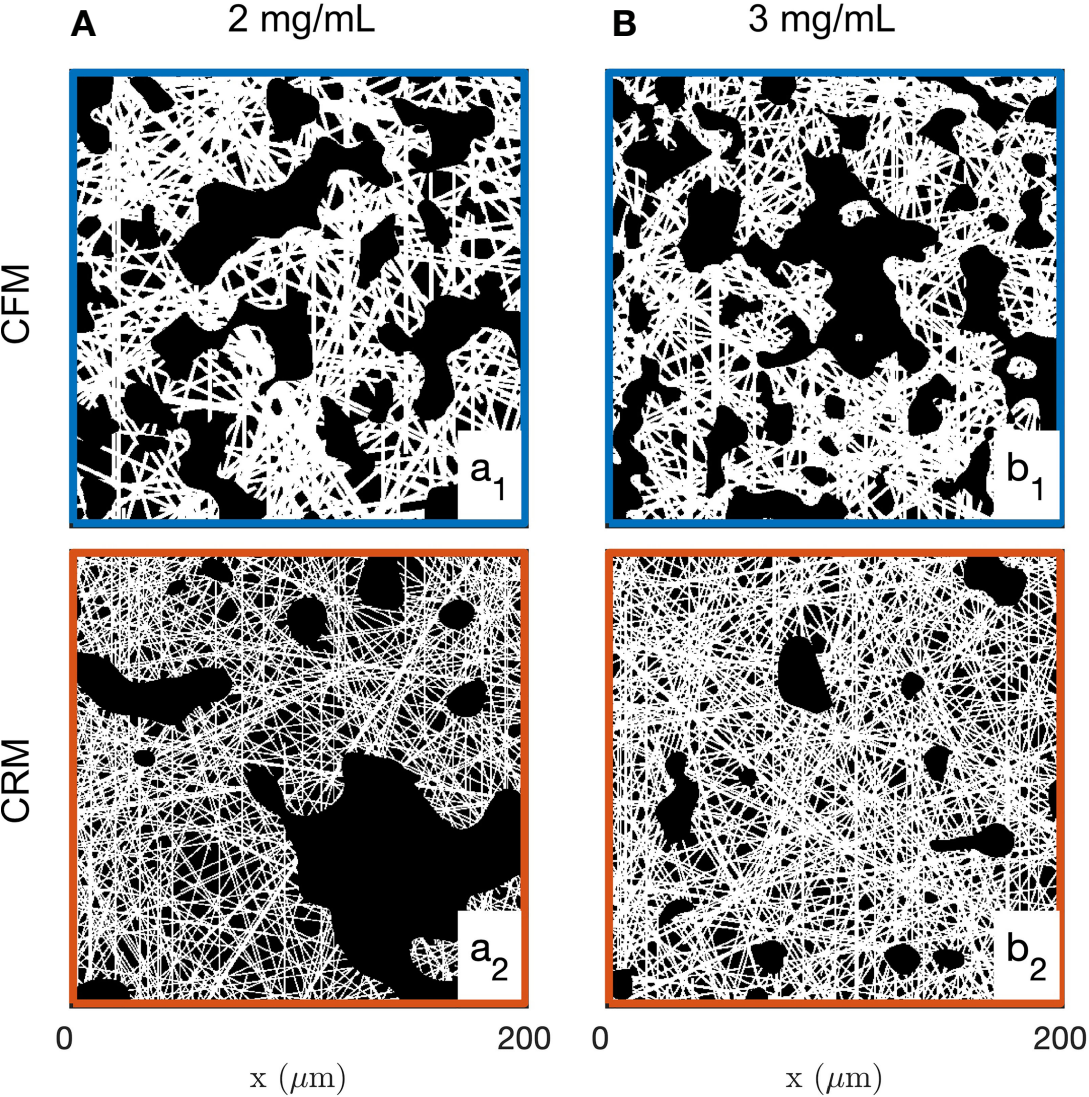
Realizations of the fibre aggregate model, for the 2 mg/mL **(a1, a2)** and 3 mg/mL **(b1, b2)** gels, based on the average values of the parameters fitted from the covariance of the CFM **(a1, b1)** and CRM **(a2, b2)** segmented images.

Realizations of the fibre aggregate model for the two gels and the two imaging modes are given in **Figure 5**. These realizations illustrate the structures captured by the covariance in the segmented images of the gels, and they largely illustrate the drawbacks of a data-analysis based on image segmentation. As mentioned earlier, the segmentation of the CFM data leads to inconsistencies between the density of the segmented images and the gel concentrations. In addition, the significant noise in the CRM data leads to unrealistically large values for the aggregate density (see **Figures 2b1, b2**, **5b1, b2**).

#### 3.2.3 Greytone model

In order to overcome the limitations of image segmentation, we generalize here the binary fibre-aggregate model so as to allow the identification of its parameters directly from grey-tone images. The binary models considered so far ignore the fact that large intensities in the images are associated with large local fibre concentrations. In the grey-tone model, we therefore assume that each fibre contributes additively (by a quantity Δ ) to the local grey-tone of the image. Namely, the image intensity where 2, 3 or more fibres overlap is 2Δ , 3Δ , etc. The model also accounts explicitly for the noisiness of the data, through uncorrelated Gaussian noise *n*(**x**) with variance *σ* ^2^ , and for a background intensity b.

These assumptions can be formally written by expressing the local image intensity at point **x** as


(9) $$I\left(\text{x}\right)=b+I_{A}\left(\text{x}\right)\times \text{Δ}\sum_{i}I_{F}^{\left(i\right)}\left(\text{x}\right)+n\left(\text{x}\right)$$

where *ℐ*_*A*_(**x**) is the indicator function of the aggregates as before, and the sum accounts for the overlapping of the fibres. In the sum, each term $I_{F}^{\left(i\right)}\left(\text{x}\right)$ is the indicator function of a homogeneous fibre model with vanishingly small number density *η* ^(*i*)^ so as to avoid the overlapping of fibres. The fibre density *ϕ*_*F*_ is then obtained again through Eq. (3), with the total number density $\eta =\sum_{i}\eta^{\left(i\right)}$ .

The statistical independence of all contributions in Eq. (9) -namely, *ℐ*_*A*_(**x**), *n*(**x**) and $I_{F}^{\left(i\right)}\left(\text{x}\right)$ for all *i*’s- enables one to calculate the corresponding grey-tone correlation function. This is done through the application of Eq. (2) to the image intensity in Eq. (9), and the result is


(10) $$C\left(r\right)={\langle I\rangle }^{2}+\eta {\text{Δ}}^{2}K_{F}\left(r\right)C_{AA}\left(r\right)+{\left(\eta \text{Δ}\right)}^{2}\left(C_{AA}\left(r\right)-\phi_{A}^{2}\right)+\sigma^{2}\delta \left(r\right)$$

where *K*_*F*_(*r*) is the geometrical covariogram of the fibres, *C*_*AA*_(*r*) is the covariance of the aggregates, the last term is the correlation function of uncorrelated Gaussian noise where *δ*(*r*) is equal to 1 if *r*=0 and to 0 otherwise, and


(11) $$\langle I\rangle =b+\eta \phi_{A}\text{Δ}$$

is the average intensity of the image.

In addition to correlation functions, the grey-tone modelling enables one to extract structural information from the grey-tone distribution itself. Because the fibres in the model are distributed according to a Boolean process, the probability for exactly *k* fibres to overlap at any point of space is a Poisson variable, namely


(12) $$\text{Prob}\left\{k\;\text{overlapping}\;\text{fibres}\right\}=\frac{\eta^{k}\exp[-\eta ]}{k!}$$

which contains Eq. (3) as a particular case, when all probabilities for *k* larger or equal to 1 are added. As each fibre contributes a quantity Δ to the local intensity, Eq. (12) can also be interpreted as the probability for observing the intensity *k*×Δ for any point inside an aggregate. Accounting also for the background intensity *b* and for the Gaussian noise, the intensity distribution is therefore


(13) $$f\left(I\right)=\left(1-\phi_{A}\phi_{F}\right)g_{\sigma }\left[I-b\right]+\phi_{A}\exp[-\eta ]\sum_{k=1}^{\infty }\frac{\eta^{k}}{k!}g_{\sigma }\left[I-\left(b+k\text{Δ}\right)\right]$$

where


(14) $$g_{\sigma }\left[x\right]=\frac{1}{\sqrt{2\pi }\sigma }\exp[-\frac{x^{2}}{2\sigma^{2}}]$$

is the centred Gaussian probability density. The quantity *f*(*I*)d*I* is the probability for a pixel to have intensity in the interval [*I*,*I*+d*I*]. In Eq. (13) the first term accounts for grey-tone distribution of the background, outside the aggregates or between fibres within the aggregates. In the second term, the sum over *k* is on the increasing number of overlapping fibres within the aggregates.

**Figure 6:**
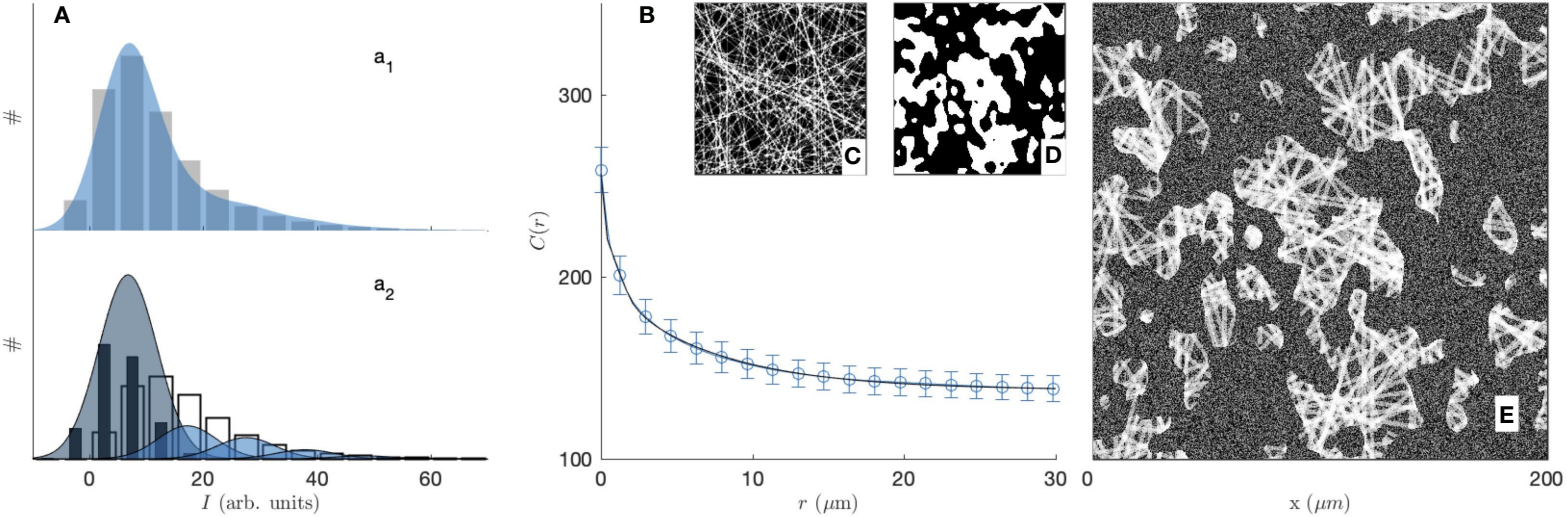
Greytone fibre-aggregates model of the 2 mg/mL type-I collagen gel, imaged by fluorescence microscopy, with **(a1)** histogram of grey levels displaying pixel counts (#) against their intensity (bars: experimental values; blue shade: model), **(B)** the correlation function (dots: experimental value; solid line: model), as well as realizations of the fibres **(C)**, aggregates **(D)** and complete model **(E)**. In **(a2)** the values inferred from the segmented images in the pores (back bars) and fibre areas (white bars) are compared with the model contributions to the background (dark shade) and increasing number of overlapping fibres (bright shades).

**Figure 7:**
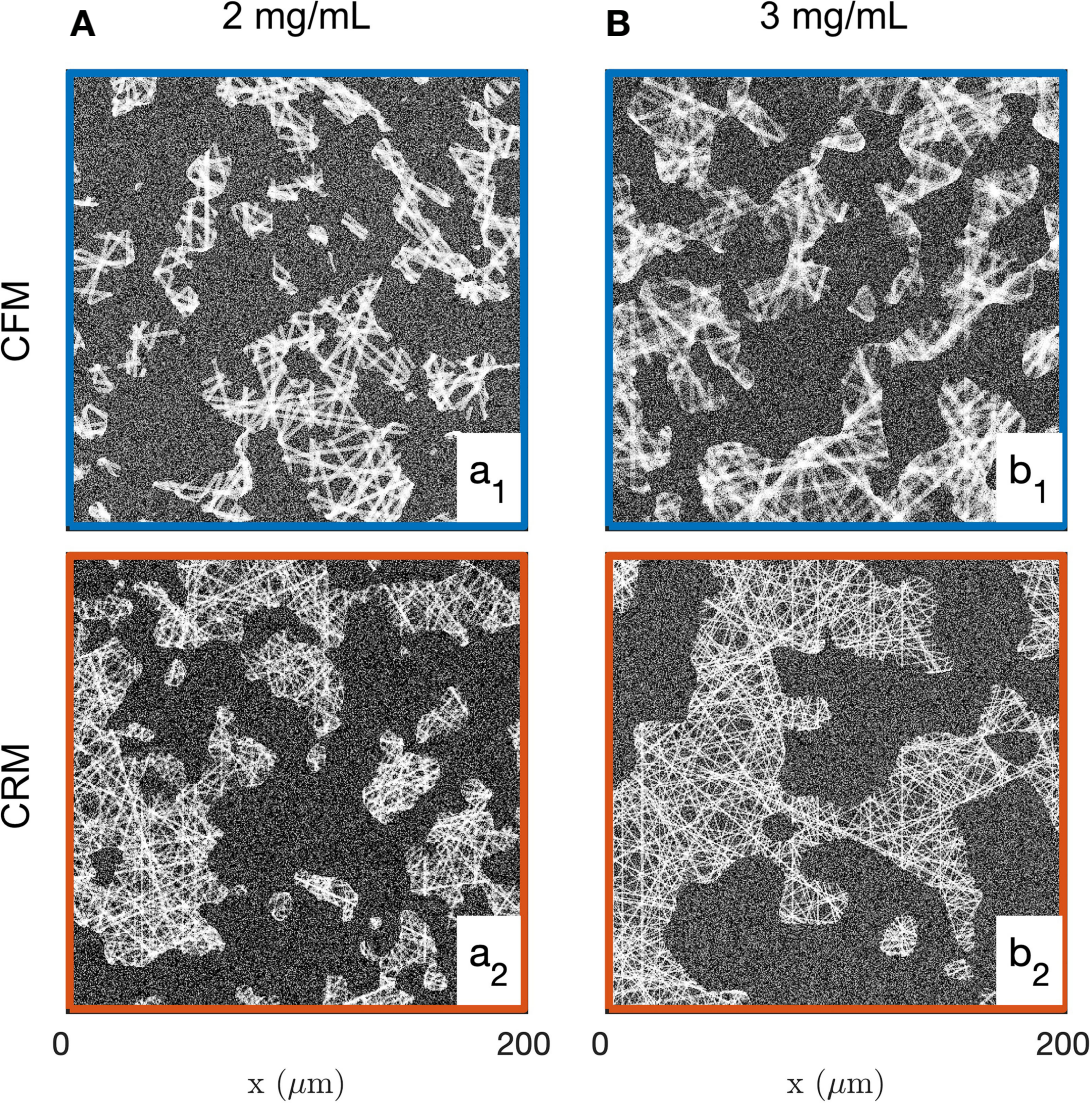
Realizations of the grey-level fibre aggregate model, for the 2 mg/mL **(a1, a2)** and 3 mg/mL **(b1, b2)** gels, based on the average values of the parameters fitted from the fluorescence **(a1, b1)** and reflectance **(a2, b2)** images.

**Figure 6** illustrates the fitting of the CRM and CFM microscopy images with the grey-tone model. The analysis is extended to the SHG imaging of the 3 mg/mL gel in the **Supplementary Material** (**Figures S4–S6**). Globally, the model captures nicely both the distribution of grey levels (**Figure 6a1**) and the correlation function (**Figure 6B**). As an independent check, **Figure 6a2** compares the modelled distribution of grey tones with the values measured in the regions of the image that were classified as pores or fibres in the segmented images (see **Figure S2**). The comparison should be considered with caution, as it is the unsuitability of the segmentation that justified the present grey-tone approach. The agreement of the two methods, however, seems reasonable. The various contributions to the fibre intensity in **Figure 6a2** also testify to the importance of fibre overlap for the image analysis. The values of the fitted parameters on the two gels and two imaging modes are reported in **Table 1**. Realizations obtained from the average values of the fitted parameters are given in **Figure 7**.

## 4 Time- and space-resolved analysis of the gel structure in the spheroid model

**Figure 8:**
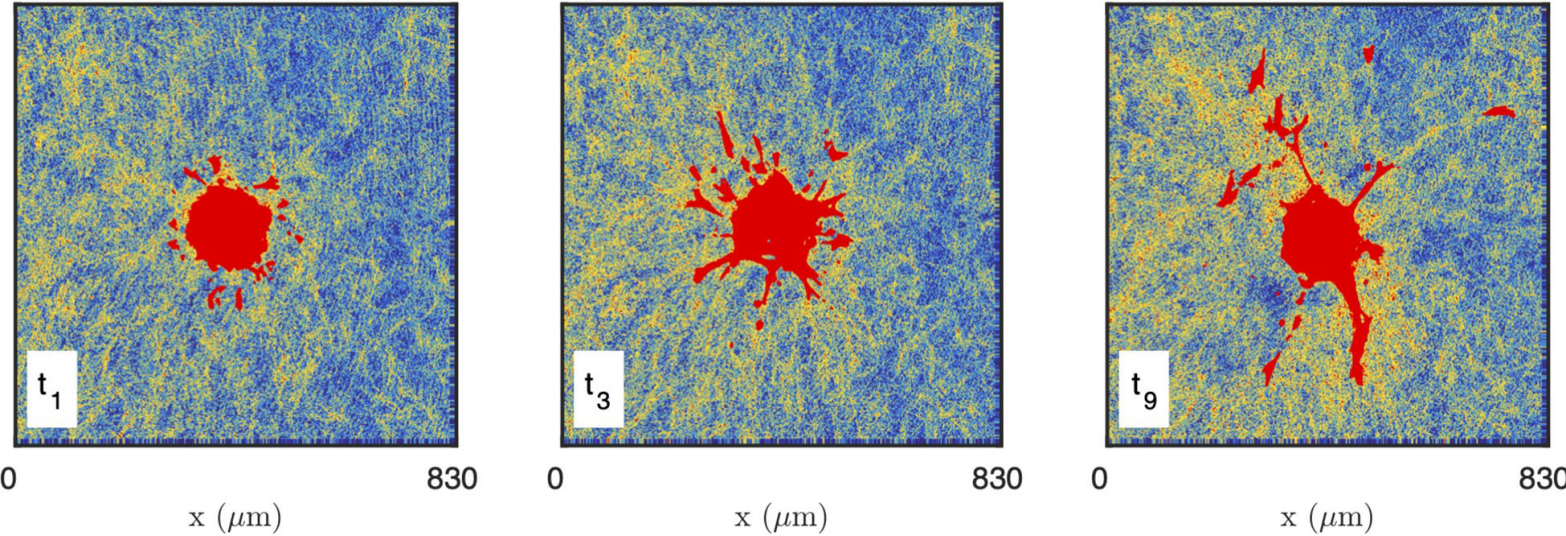
Images of the CAF spheroid and surrounding gel at various times: t1 = 30 min, t3 = 210 min, t9 = 750 min. The images correspond to one height z cutting through the spheroid, with the cells shown in red and the density of the gel coded from blue (low density) to yellow (high density).

As the presence of fluorescently-labelled collagen has been shown to interfere with the polymerization process when performed at a low temperature (69), unlabelled collagen (2 mg/mL) was used and CRM was selected when performing time-lapse imaging of collagen-embedded spheroids. Time-resolved reflectance images of the platelet-derived growth factor-BB (PDGF-BB)-treated CAF spheroid and surrounding gel are shown in **Figure 8**, for times ranging from 30 min to 750 min. In order to characterize the spatiotemporal dynamics of cell interaction with the surrounding collagen matrix during cell migration, the system was imaged at lower resolution than the acellular gels considered so far. At the scale of the spheroid, individual fibres are not visible and the only structures detected in the gels are the aggregates. The images testify to a progressive local densification of the gel structure close to the spheroid and migrating cells but it is unclear whether other structural characteristics of the gel evolve as well. Moreover, as the gel heterogeneity seems to develop at the same scale as the spheroid, it is difficult to ascertain whether the effect of the cells is through direct contact with the gel, or if long-range effects are also operative.

**Figure 9:**
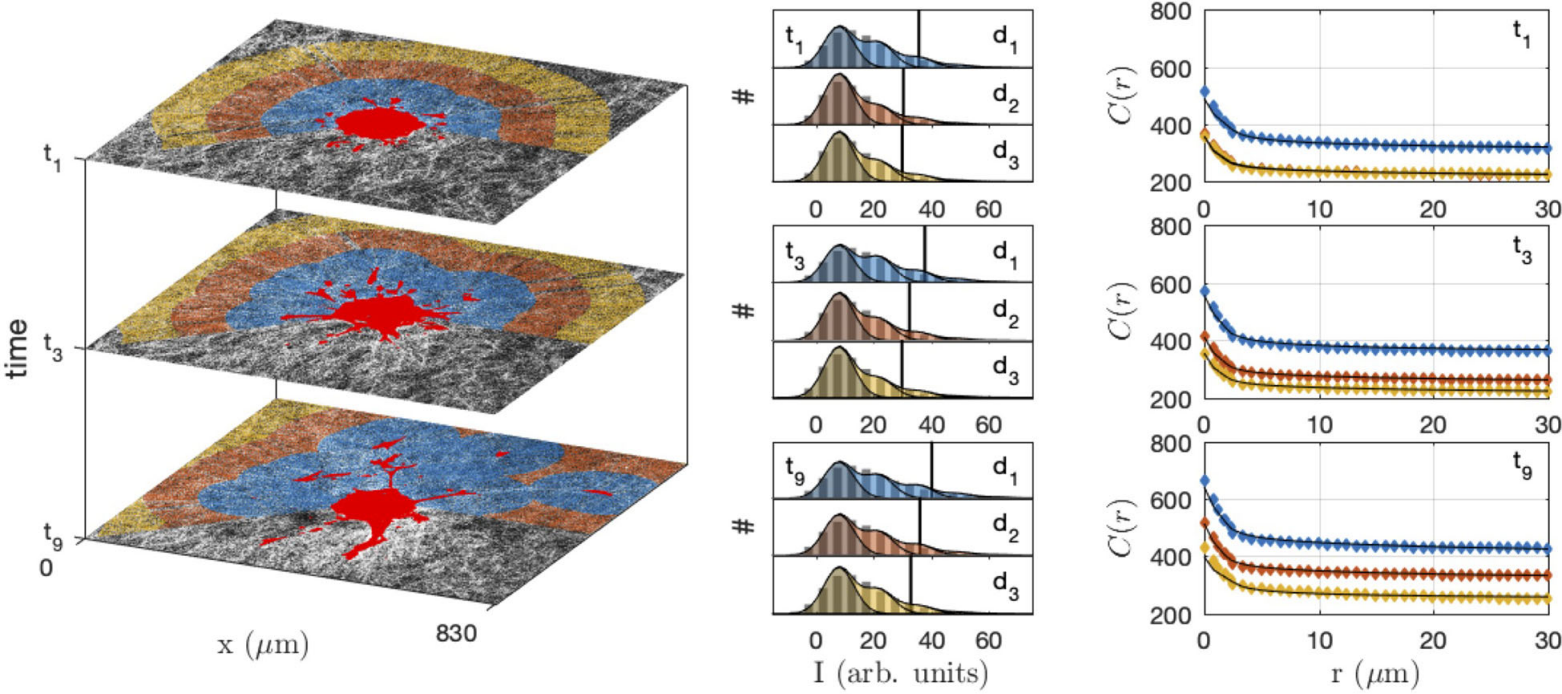
Collagen structure analysis in the neighbourhood of the CAF spheroid, at different times (*t*_1_ = 30 min, *t*_3_ = 210 min, *t*_9_ = 750 min) and distances from the cells (*d*_1_< 100 *µ*m, 100 µm< *d_2_* < 200 µm and 200 µm< *d_3_* < 300 µm). In the original images (left) the gel is shown in grey and the cells in bright red, and the same colour code is used for the distances in the grey-tone distributions (middle) and correlation functions (right). The middle panel displays the histogram of grey-levels (pixel counts # against their intensity I), with the bars being the experimental distributions, etc. The shaded areas are the grey-tone model, with the background contribution (darker) and increasing number of fibre overlaps. In the right panel the grey-tone correlation functions *C*(*r*) are shown, with the dots being the experimental values and the solid lines being the grey-tone model.

In order to quantitatively investigate the modification of the gel structure in relation with cell migration, the gel area surrounding the spheroid was decomposed into layers corresponding to increasing distances to the closest cells (shown as different colors in **Figure 9** left). At each time step, the layers were recalculated according to the updated position of the cells. The shapes of the equal-distance layers become increasingly complex with time, as a consequence of the irregularity of the cell invasion pattern. The histogram of grey tones and the correlation function of the collagen were measured within each layer at each time step (**Figure 9** middle and right).

The densification of the fibres in the vicinity of the spheroid as well as of the migrating cells is manifest in **Figure 9** through the progressive shifting of the grey-tone distributions towards brighter values (middle panel), and notably the 90% percentile (vertical lines). A similar evolution is observed in the correlation functions that progressively shift towards larger values with time. The evolution is quite rapid for the collagen in direct contact with the cells (d_1_) and much slower for larger distance (d_3_).

**Figure 10:**
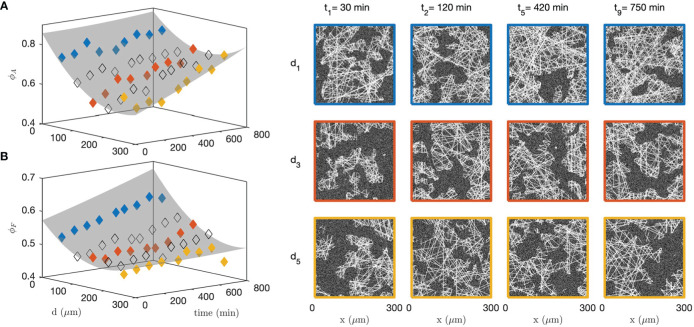
Fitted collagen structure for various times and distances to the CAF cells, with aggregate density *ϕ*_*A*_ **(A)** and fibre density within the aggregates *ϕ*_*F*_ **(B)**. The grey surface is a guide to the eye. Specific realizations are shown on the right panel to illustrate the significance of the parameters. The colour code for distances is the same as in **Figure 9**; the non-colored symbols point to intermediate distances not shown in **Figure 9**.

In order to analyse the structural modifications corresponding to the changes in grey-tone distributions and correlation functions, all the data measured at 9 successive time steps and 5 different distances from the cells were fitted simultaneously with the grey-tone model (see **Figure 9** for 3 times and 3 distances). Among the parameters of the grey-tone model, the densities and sizes bear a structural meaning (see **Table 1**), but other parameters merely characterize the imaging mode. This is notably the case for the fibre contrast Δ , the background intensity *b* , and the noise intensity *σ* (see Eqs. 10 and 13). For the fitting of the data, different values of the structural parameters were allowed for each time and distance, but a unique value of the imaging parameters was allowed for all images. This is justified as the time series images of the gel and spheroid were measured through a time-lapse protocol with unchanged imaging parameters. The values of the imaging parameters from the fit are *b*≃9 , Δ≃15 and *σ*≃5. The structural parameters are *D*_*F*_≃2 µm and *L*_*A*_≃19 µm for all distances and times, and the volume fractions *ϕ*_*A*_ and *ϕ*_*F*_ are as shown in **Figures 10A, B**.

**Figure 10** testifies to qualitatively different structural changes in the gel in direct contact with the CAF cells and distant from them. Close to the cells (blue series), a large number of aggregates have already formed before the first measurement time ( *t*<30 min). It remains relatively constant afterward with density *ϕ*_*A*_≃80*%*, which can also be described in terms of a matrix porosity that remains constant around 20%. In the meantime, the fibre density within the aggregates increases steadily all throughout the experiment. This contrasts with the situation far from the cells (yellow series), where the aggregate density *ϕ*_*A*_ increases far more progressively and the fibre density within the aggregates *ϕ*_*F*_ remains altogether constant. These qualitatively different structural changes close and far from the cells are illustrated with right panel of **Figure 10** for various distances and times.

## 5 Discussion

The structure of collagen gels is a highly hierarchical one (20). Starting from molecular dimensions, the smallest structures are the tropocollagen triple helices (diameter 1.5 nm), assembled into protofibrils and fibrils (6 to 100 nm), woven into fibres (1 µm), which are eventually connected to form the gel matrix. The matrix, however, is not homogeneous at a scale larger than 10 µm, as visible in all images of **Figure 1**. A central structure of collagen at large scale is the presence of fibre aggregates, which can also be thought of in terms of pores in the matrix. The latter are the most salient structures in the gels at the scale of the cells (**Figure 8**), and it is therefore natural to enquire how they are modified by neighboring cells. We addressed this question by developing first a novel image analysis methodology to characterize the large-scale structure of collagen, by validating it on two acellular gels, and finally applying it to characterise the evolution of the collagen structure surrounding a spheroid of CAF cells.

The characterisation of the disordered pore-and-aggregate structure of the gels is challenging, and calls for a statistical description. The approach we explored here is based on a mathematical modelling, which aims at statistically capturing the main characteristics of the gels through a small number of structurally meaningful parameters. It is important to stress that the goal of the models is not to reproduce a local gel structure, as one would typically observe in a single image, but also to capture the structure variability. This feature is essential to assign a single value to the structural parameters based on many different images of one and a single gel.

In that spirit we first considered a homogeneous fibre model (**Figure 3**). This simple model was found to be incompatible with the experimental covariances of the collagen, which statistically confirmed the presence of fibre aggregates and pores. We then proposed a two-scale model, where the small-scale structure is described as a homogeneous model of random fibres, and the large-scale aggregate structure is obtained by using a clipped Gaussian-field to carve pores out of it (**Figure 4**). In this two-scale model, two parameters *ϕ*_*A*_ and *L*_*A*_ describe the density and size of the aggregates, and two parameters *ϕ*_*F*_ and *D*_*F*_ describe the density and the diameter of the fibres inside the aggregates. Analytical expressions were derived to fit experimental characteristics of the gels measured from the images, and infer the values of the mentioned structural parameters.

A classical image-analysis method consists in segmenting first the images to identify objects of interest and subsequently measuring them. This approach was proved unsuitable in the present context, as segmentation would result in the loss of valuable structural information. The collagen-rich areas of the gels are indeed characterized by broad grey-tone distributions (see **Figure S2b**), with large intensities being associated with the close proximity or overlapping of many fibres. Image segmentation would amount to treating all these regions on the same footing, and would therefore bias any measurement.

We developed a method to identify the model parameters directly from grey-tone microscopy images, through the fitting of the covariance function and histogram of intensities. The covariance -or correlation function, in the case of grey-tone images- is a very informative structural descriptor (95, 100) that can easily be measured on any image through fast Fourier transforms. Covariance analysis offers a variety of advantages. First, it provides a robust and fully objective statistical description of the spatial distribution of the objects that make up an image. This is particularly useful for structures as complex and disordered as fibrillar collagen gels. Second, it reduces the impact and possible bias of image preprocessing, which is absent altogether in the case of grey-tone correlation functions. Finally, it also provides an efficient way to cope with noisy images, as noise is typically defined as the non-correlated contribution to an image.

Globally, the procedure based on grey-tone measurements (correlation functions and intensity distributions) and on their modelling proved to be quite consistent with respect to the two types of imaging modes considered in the paper. The absolute values of the parameters obtained through CRM and CFM data differ slightly, but identical trends are detected when comparing the 2 mg/mL and 3 mg/mL acellular gels (**Table 1**). The overall fibre density *ϕ*_1_ is larger in the 3 mg/mL gel, as it should. Our analysis shows that this is due largely to an increased density of aggregates *ϕ*_*A*_ while the fibre density within the aggregates *ϕ*_*F*_ is almost identical in the two gels. The characteristic size of the aggregates *L*_*A*_ is larger in the 3 mg/mL gel because the aggregates are more numerous and form a larger connected structure. To further highlight the importance of using grey-tone images, we also performed the same analysis on segmented images. In that case, segmentation leads to the undesirable situation where the density of the segmented images is opposite to the known collagen concentration of the gels in the case of fluorescence data (**Table 1** and **Figure 2A**).

From a methodological point of view, the application of the grey-tone model to analyze the structure of the 2 mg/mL and 3 mg/mL gels in both CRM and CFM modes, validated the overall image analysis approach and enabled us to apply it to the dynamic model of CAF spheroid invasion (**Figures 9**, **10**). Matrix remodelling has been widely documented in earlier works (63, 101–104). Contractility of collagen-embedded cells generates strains on the collagen fibres, leading to their local densification (19, 105) and reorganization (102, 103, 106). This process is especially evident for cells of mesenchymal origin such as CAFs, which are characterized by high expression levels of the contractile myosin protein and collagen-binding integrins (105). In our experimental model, CAF spheroids were treated with PDGF-BB, a growth factor known to stimulate collagen gel contraction (107, 108). By pulling on collagen fibers, cells stiffen their microenvironment and induce irreversible collagen deformations, thereby altering locally the mechanical properties of the ECM. This remodelled microenvironment in turn provides guidance cues for the nearby cells through durotactic and/or topotactic migrations (109–111). Besides these guidance cues, high concentrations of fibrillar collagen also promote the local invasion of cells by inducing the formation of specialized cellular extensions termed invadopodia implicated in the local proteolytic remodeling of the ECM (112).

Our use of a two-scale stochastic model to describe the collagen structure and the identification of its parameters from grey-tone images, enables us to describe the ECM remodelling in more detail than simply the collagen density. The pristine state of the ECM in the CAF spheroid model is the one observed at early times *t* and large distances *d* from the cells. The relevant values of the aggregate and fibre densities are *ϕ*_*A*_≃0.6 and *ϕ*_*F*_≃0.5 based on **Figures 10A, B**. These values are reasonably consistent with those of the 2 mg/mL acellular gels in **Table 1**, and the slight differences can be attributed to the lower resolution used in the time-resolved analysis of the spheroids. Starting from that initial state, our space- and time-dependent analysis of the collagen structure shows that the ECM densification happens *via* two distinct mechanisms, namely: the increased density of fibre aggregates *ϕ*_*A*_ and the increased fibre density *ϕ*_*F*_ within the aggregates. The two mechanisms are both at work in the gel in close contact with the cells. The aggregate density increases very fast, from about *ϕ*_*A*_≃60% to 80% in less than 30 mins (**Figure 10A**), similar to the cancer cell spheroids investigated by Chen et al. (106) which exert a strong contraction of the surrounding collagen immediately after embedding in the matrix. The fibre densification also takes place inside the aggregates in a much more progressive way, as it occurs over hours (**Figure 10B**).

The global densification of the gel resulting from the two processes is quite significant, as the overall fibre density in contact with the cells - estimated as *ϕ*_1_=*ϕ*_*A*_×*ϕ*_*F*_ - approximately passes from *ϕ*_1_≃ 30% to 50%. It is important to stress that this only concerns the fraction of fibres that are visible in our experiments, which is determined by the imaging technique (CRM vs CFM) and by the spatial resolution of the imaging system (∼ 0.59 µm/pixel in our case). As smaller fibrils and protofibrils are undetected at the considered scale, the apparent densification does not contradict the fact that the total collagen concentration has to remain locally constant. We also cannot exclude that a fraction of the aggregate densification observed by CRM in our spheroid model results from a CAF-mediated partial reorientation of the more vertical collagen fibres relative to the imaging plane (113). In any event, the analysis testifies to significant yet different remodelling of the gel structure at two scales simultaneously.

Interestingly, the remodelling of the collagen is not limited to the regions in direct contact with the cells. Significant structural changes are observed also in regions as far as 300 µm away from the closest CAF cell, but the phenomenology is distinctly different there. In those regions, the density of fibre aggregates increases slowly, over hours, while the fibre density within the aggregates remains constant. Similar long-range (up to 1300 µm) mechanical signals generated by fibroblasts have been shown to propagate through fibrillar collagen networks. Such mechanical cues can be sensed far beyond the signal source by cells sharing the same substrate, which is responsible for the ability of contractile fibroblasts to induce the migration of macrophages towards the force source (62).

From a methodological point of view, it is important to stress that the discrimination between the two types of densification processes is robust. Considering **Figure 6A**, an increase of aggregate density *ϕ*_*A*_ with constant fibre density *ϕ*_*F*_ would be manifest through a reduction of the background contribution (dark blue) in favor of the fibre contribution (bright blue) but the shapes of the two contributions would remain unchanged. By contrast, any increase in the fibre density *ϕ*_*F*_ is accompanied by an increasing number of pixels where the fibres overlap, which would necessarily extend the grey-tone distribution towards higher intensities. With that in mind, the observed shifting of the 90%-percentile of the image intensity in **Figure 9** can only be interpreted as an increase of *ϕ*_*F*_ , independently of any evolution of the aggregate density *ϕ*_*A*_. As for the grey-tone correlation function, its two asymptotic values for *r*=0 and *r*→*∞* depend only on the total fibre density *ϕ*_1_=*ϕ*_*A*_×*ϕ*_*F*_. The combination of the grey-tone distribution and correlation function is therefore key to discriminate the two different densification processes.

## 6 Conclusion

In this study, we developed a novel and multidisciplinary image analysis approach to investigate the remodelling of fibrillar collagen in a 3D spheroid model of cellular invasion. Unlike existing work, most of which focus on fibre densification of the collagen network and on small-scale fibre reorientation, we focused here on the structural modification of the collagen matrix at the scale of a few microns, comparable with that of the cells. This was achieved by first developing a novel image analysis method based on the stochastic modelling of the acellular gel structure, and applying it afterwards to study the space- and time-dependent reshaping of the collagen matrix by migrating CAFs.

The analysis of acellular collagen gels (without embedded cells) investigated by confocal microscopy in both reflectance and fluorescence modes, shows that the structure of the gels is not homogeneous at the scale of about 10 microns. The structure consists in regions with high fibre density separated by depleted regions, which can be thought of as fibre aggregates and pores. In order to mathematically describe this structure, we developed a two-scale stochastic model with a clipped Gaussian-field model for the aggregates and pores, and a homogeneous Boolean model to describe the fibre network within the aggregates. We also developed a method to identify the model parameters from the grey-tone distributions and correlation functions of the gel images. The specificity of the method is that it applies to the unprocessed grey-tone images, and it can therefore be used with noisy time-lapse CRM images of non-fluorescent collagen.

When applied to the collagen-embedded CAF spheroid images, the method we developed testifies that the invasion of PDGF-BB-treated CAFs is accompanied by an overall densification of the collagen gel while the sizes of the pores and aggregates, as well as that of the fibres, remains largely unchanged. Interestingly, the densification occurs differently for the gel in direct contact with the cells or far away from them. The gel in close contact with the invading cells densifies through the rapid increase of the number of aggregates, over less than 30 min, followed by the slow increase of fibre density within the aggregates, over hours. By contrast, the densification occurring in the gel located farther away from the cells occurs *via* the slow increase of the aggregate density, while the density of fibres within the aggregates remains constant.

At the present stage, one can only speculate on the biomechanical mechanisms responsible for the two-scale densification. The very observation of two distinct phenomenologies hint at diverse mechanisms, which presumably involve both biochemical and mechanical effects.

## Data availability statement

The original contributions presented in the study are included in the article/**Supplementary Material**. Further inquiries can be directed to the corresponding authors.

## Author contributions

AN and EM designed the biological experiments; CG and SB designed the mathematical analysis; IB prepared the biological samples; TL performed the confocal microscopy imaging; CG developed the mathematical models, analyzed the data and prepared all figures; CG, EM, SB, and AN wrote the manuscript. All authors contributed to the article and approved the submitted version.

## Supplementary material

Supplemental data A Supplementary Material file is available with (i) the expression profile of 7 CAF markers in MMTV-PyMTderived CAFs and epithelial cancer cells, (ii) details about the method used to segment the gel images into fibre and background pixels, (iii) a description of the Gaussian Random Field approach used to model the fibre aggregates, (iv) a comparison of the reflectance (CRM) and second-harmonic generation (SHG) results for the 3 mg/mL acellular gel.
