# Supplementary material for: Remodelling of the fibre-aggregate structure of collagen gels by cancer-associated fibroblasts: A time-resolved grey-tone image analysis based on stochastic modelling
Source: Front Immunol. 2023 Feb 3;13:988502. doi: 10.3389/fimmu.2022.988502 (PMC9936192; doi:10.3389/fimmu.2022.988502)
Supplement: Supplemental data — A Supplementary Material file is available with (i) the expression profile of 7 CAF markers in MMTV-PyMTderived CAFs and epithelial cancer cells, (ii) details about the method used to segment the gel images into fibre and background pixels, (iii) a description of the Gaussian Random Field approach used to model the fibre aggregates, (iv) a comparison of the reflectance (CRM) and second-harmonic generation (SHG) results for the 3 mg/mL acellular gel. [file DataSheet_1.pdf]

Supplementary Material

1 CHARACTERIZATION OF THE MMTV-PyMT-DERIVED CAF AND EPITHELIAL CANCER CELLS

CAFs and epithelial cancer cells were isolated from spontaneous mammary tumors arising in MMTV-PyMT female mice and the expression of 7 CAF's markers, including fibroblasts activation protein-alpha (FAP), actin alpha 2 (ACTA2), tenascin C (TNC), collagne type I (COL1), collagen type III (COL3), platelet derived growth factor receptor beta (PDGFRB) and integrin alpha 11 (ITGA11), was analyzed by reverse transcription quantitative polymerase chain reaction (RT-qPCR).

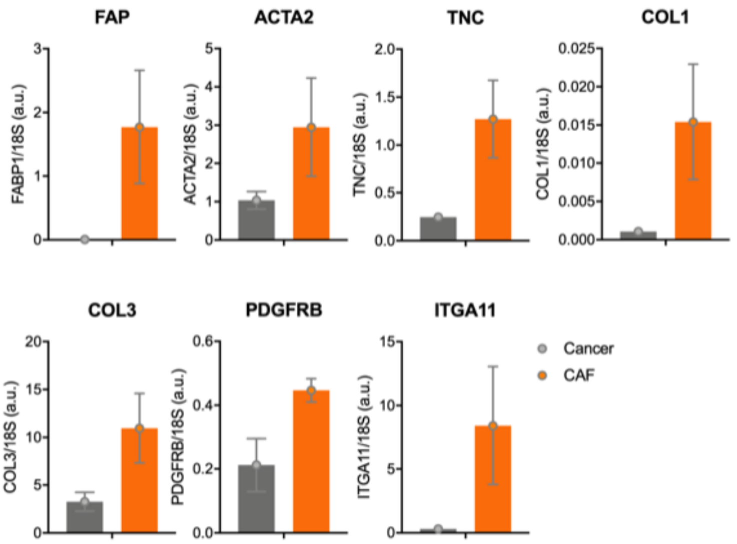

Figure S1. RT-qPCR analysis of the mRNA levels of 7 markers of CAFs in MMTV-PyMT-derived CAFs (orange boxes) and epithelial cancer cells (grey boxes). The mRNA levels were normalized by the abundance of the 18S rRNA. Mean ±SEM of 3 and 2 independent isolations of epithelial cancer cells and CAFs, respectively.

2 SEGMENTATION OF THE GEL IMAGES

When necessary, the method in Fig. S2 was used in the main text for segmenting the grey-tone images of the acellular collagen gels into black-and-white images.

Two distinct thresholds, I_1 and I_0, are extracted from the histogram of grey tones in the background-corrected images by extrapolating the linear slope at the inflection point of the cumulated distribution (Fig. S2b). Pixels in the image brighter than I_1 and darker than I_0 are assigned to the fibres (Fig. S2c_1) or pore space (Fig. S2c_2), respectively. This leaves a large fraction of the pixels unassigned, neither to the fibres nor to the background. For the those pixels, a distance criterion is applied: any pixel closer to a previously segmented fibre than to any pore is classified as a fibre, and vice versa (Fig. S2e). The distance from any

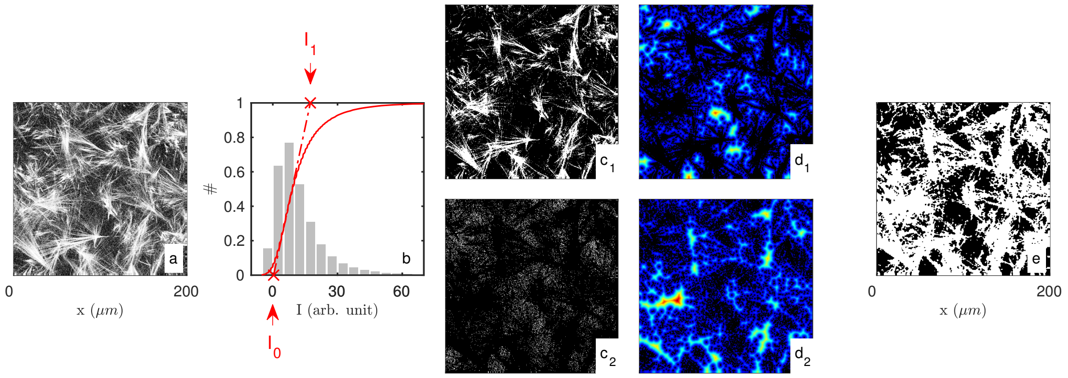

Figure S2. Segmentation of the gel images, with (a) the background-corrected grey-tone image, (b) the corresponding intensity distribution, with the cumulated curve (in red) used to determine two thresholds I_0 and I_1 ($\times$), (c) the segmented brightest fibres (top, $I > I_1$) and darkest pores (bottom, $I < I_0$), (d) the distance map to the closest bright fibre (top) or dark pore (bottom), and (e) the segmented image with fibre aggregates in white.

pixel of the image to the brightest fibres ($I > I_1$) and darkest pores ($I < I_0$) are shown in Figs. S2d$_1$ and S2d$_2$.

The entire segmentation procedure was designed to avoid any manual input from the user (e.g. thresholds, etc.), and thereby ensures its complete objectivity.

3 GAUSSIAN-FIELD MODEL OF FIBRE AGGREGATES

The shape of the fibre aggregates is modelled in the main text as a clipped Gaussian random field. This model can be thought of as being built in the following two steps. First, a Gaussian random field $W(\mathbf{x})$ is created, *e.g.* as a superposition of a large number N of sine waves as

$$W(\mathbf{x}) = \sqrt{\frac{2}{N}} \sum_{n=1}^{N} \sin\left[\mathbf{q}_n \cdot \mathbf{x} - \varphi_n\right] \tag{S1}$$

where the wavevectors $\mathbf{q}_n$ are isotropically oriented and their moduli are drawn from a user-specified distribution $f_W(q)$. From a mathematical point of view, specifying $f_W(q)$ is equivalent to specifying the correlation function of the field

$$g_W(r) = \langle W(\mathbf{x}) W(\mathbf{x} + \mathbf{r}) \rangle \tag{S2}$$

as the two quantities are Fourier transforms of each other. Based on earlier work, we chose the specific analytical form

$$g_W(r) = 1/\cosh\left[r/L_A\right] \tag{S3}$$

where the length L_A is a parameter of the model. A realization of such a Gaussian field with $L_A = 10\mu$m is shown in Fig. S3a.

As a second step of the modelling, the Gaussian field is clipped at a user-chosen threshold α. In the case of the main text, this corresponds to modelling the aggregates as the points of space where $W(\mathbf{x})$ takes values larger than α. The parameters α controls the volume fraction of the aggregates through

$$\phi_A = \frac{1}{2}\left(1 - \text{erf}\left[\alpha/\sqrt{2}\right]\right) \tag{S4}$$

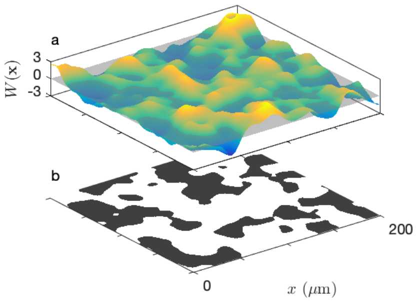

Figure S3. Clipped Gaussian random field model, whereby a Gaussian field $W(\mathbf{x})$ (a) is converted to a black-or-white structure (b) through a clipping procedure. In this case, the threshold is set to $\alpha = 0$ corresponding to volume fraction $\phi_A = 0.5$.

where erf is the error function. The covariance is calculated as

$$C_{AA}(r) = \phi_A^2 + \frac{1}{2\pi}\int_0^{\mathrm{asin}[g_W(r)]} \exp\left[-\alpha^2/(1+\sin(t))\right] \mathrm{d}t \tag{S5}$$

with the function $g_W(r)$ given in Eq. (S3).

4 COMPARISON WITH SECOND-HARMONIC GENERATION (SHG) IMAGING OF COLLAGEN FIBERS

For the sake of comparison, the structure of the same 3mg/mL collagen gel as discussed in the main text was also investigated by Second-Harmonic Generation (SHG) microscopy. SHG occurs when two photons interact with optically non-linear material and merge to generate a new photon with twice the energy and half the wavelength of the initial photons. Fibrillar collagen non-linear optical response, which results in a strong SHG signal, originates from its noncentrosymmetric triple helical molecular assemblies, which exhibit large hyperpolarizabilities.

An example of the SHG images of the 3mg/mL gel is given in the inset of Fig. S4a, together with the grey-tone correlation function calculated from a total of 15 images similar to the one shown in the figure. To ease the comparison, the CRM data of the same gel (as discussed in the main text) is provided in the right panel of the figure. Globally, the two imaging techniques testify to the presence of qualitatively similar structures, consisting in aggregated collagen fibers separated by empty regions. The images from SHG microscope are much more contrasted with a better signal-to-noise ratio than from the CRM microscope. As we discuss in the main text, only the latter can be used in our experimental context for the time-lapse monitoring of the collagen remodelling. We here show how the grey-tone modelling of the images enables one to extract the same structural information from the two types of images, in spite of the different contrasts and signal-to-noise ratios.

The fitting of the grey-tone model to the SHG and CRM images of the 3mg/mL gels are illustrated in Figs. S5 and S6, respectively. The grey tones extend over different ranges in the two imaging techniques (Figs. S5a and S6a) and the values of the correlation functions are accordingly distinctly different (Figs. S5b and S6b). In spite of the differences of the grey-tone statistics in the two types of images, the structures

Table S1. Comparison of CRM and SHG results: imaging and structural parameters, obtained through the grey-tone fibre-aggregate model from the grey-tone histogram and correlation functions from Figs. S5 and S6.

Imaging	b (-)	Δ (-)	σ_n(-)	ϕ_A (-)	L_A (μm)	ϕ_F (-)	D_F (μm)
CRM	7.2 ± 3	16 ± 1	7.3 ± 5	0.57 ± 0.02	7.9 ± 0.4	0.61 ± 0.02	0.49 ± 0.02
SHG	4.3 ± 7	19 ± 3	8.5 ± 3	0.47 ± 0.07	7.3 ± 1.2	0.92 ± 0.03	0.9 ± 0.3

b: background intensity; Δ: contrast; σ_n: noise amplitude; ϕ_A, L_A: density and size of the aggregates; ϕ_F, D_F: density and diameter of the fibres. The error bars are the standard deviations observed from the fits of the fifteen images in each condition.

identified by the grey-tone model are relatively consistent. This is visible in the realizations shown in Figs. S5e and S6e.

The similarity of the collagen structures detected by the two microscopy techniques is further confirmed by the values of the fitted parameters (See Tab. S1). The consistency is quite good for the characteristics of the aggregates (density ϕ_A and size l_A), which are the main structures visible at the scale of the images. Perhaps not surprisingly, the smaller-scale inner structure of the aggregates (fiber density ϕ_F and diameter D_F) is found to be more dependent on the microscopy technique. Globally, the model enables to capture the main structural characteristics of the collagen gels at the considered scale, independently of the imaging technique.

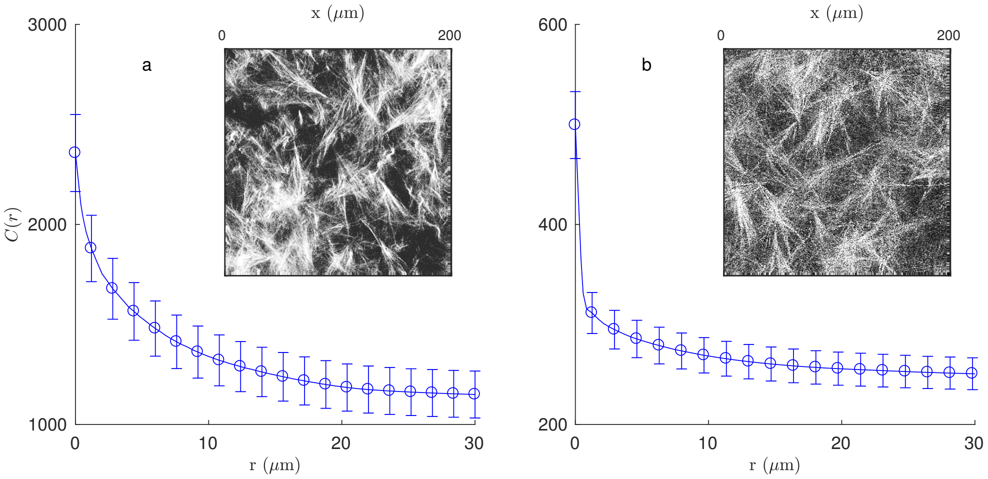

Figure S4. Grey-tone correlation functions of the 3mg/mL collagen gel, obtained from second-harmonic (left) and reflectance (right) images. The inset is one of the fifteen images used for the calculations. The right panel is identical to Fig. 2d$_2$ of the main text. SHG images were acquired with a confocal multiphoton laser scanning microscope (A1R MP+, Nikon) with a 25x/1.1 N.A. water immersion objective (Nikon). The resulting images have voxel size of 0.4025$\times$0.4025$\times$0.4 μm^3.

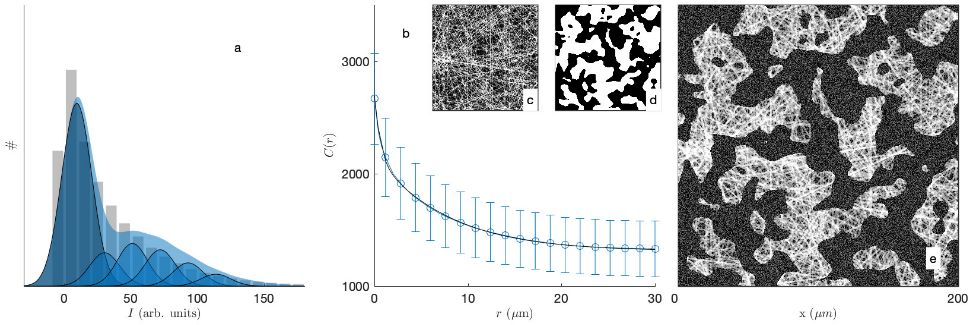

Figure S5. Fitting of the grey-tone histogram (a) and correlation functions (b) of the 3 mg/mL collagen gel, from second-harmonic generation (SHG) microscopy images. A realization of the fitted model is shown in (e) with the fiber and aggregate contributions in (c) and (d).

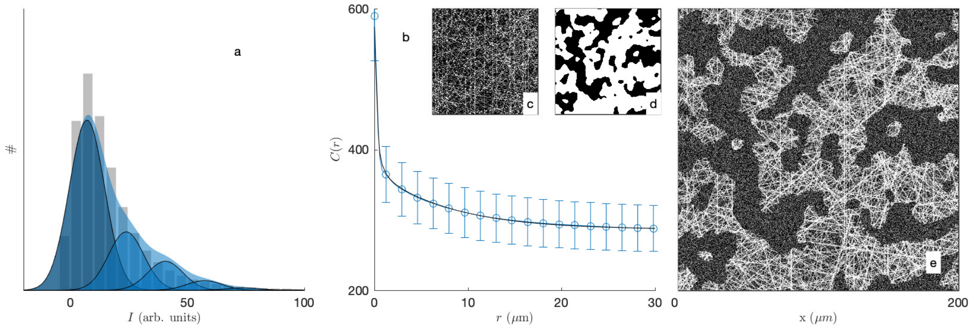

Figure S6. Fitting of the grey-tone histogram (a) and correlation functions (b) of the 3 mg/mL collagen gel, from reflectance (CRM) microscopy images. A realization of the fitted model is shown in (e) with the fiber and aggregate contributions in (c) and (d).
